# Multi-Aperture Shower Design for the Improvement of the Transverse Uniformity of MOCVD-Derived GdYBCO Films

**DOI:** 10.3390/ma10091088

**Published:** 2017-09-15

**Authors:** Ruipeng Zhao, Qing Liu, Yudong Xia, Fei Zhang, Yuming Lu, Chuanbing Cai, Bowan Tao, Yanrong Li

**Affiliations:** 1 State Key Lab of Electronic Thin Films and Integrated Devices, University of Electronic Science and Technology of China, Chengdu 610054, China; zhaoruipeng1990@sina.com (R.Z.); liuqing0435@gmail.com (Q.L.); yrli@uestc.edu.cn (Y.L.); 2 School of Physical Science and Technology, Southwest Jiaotong University, Chengdu 610031, China; ydxia@swjtu.edu.cn; 3 Chengdu Fine Optical Engineering Research Center, Chengdu 610041, China; zfchunri@163.com; 4 School of Physics, Shanghai University, Shanghai 200444, China; ymlu@staff.shu.edu.cn (Y.L.); cbcai@shu.edu.cn (C.C.)

**Keywords:** multi-aperture shower, GdYBCO, MOCVD, morphology, critical current

## Abstract

A multi-aperture shower design is reported to improve the transverse uniformity of GdYBCO superconducting films on the template of sputtered-LaMnO_3_/epitaxial-MgO/IBAD-MgO/solution deposition planarization (SDP)-Y_2_O_3_-buffered Hastelloy tapes. The GdYBCO films were prepared by the metal organic chemical vapor deposition (MOCVD) process. The transverse uniformities of structure, morphology, thickness, and performance were characterized by X-ray diffraction (XRD), scanning electron microscopy (SEM), step profiler, and the standard four-probe method using the criteria of 1 μV/cm, respectively. Through adopting the multi-aperture shower instead of the slit shower, measurement by step profiler revealed that the thickness difference between the middle and the edges based on the slit shower design was well eliminated. Characterization by SEM showed that a GdYBCO film with a smooth surface was successfully prepared. Moreover, the transport critical current density (*J_c_*) of its middle and edge positions at 77 K and self-field were found to be over 5 MA/cm^2^ through adopting the micro-bridge four-probe method.

## 1. Introduction

High performance REBa_2_Cu_3_O_7−δ_ (REBCO, RE: rare earth elements) high-temperature superconducting (HTS)-coated conductors have been successfully prepared for the applications of electric power, such as superconducting transmission cables, superconducting motors, superconducting generators, superconducting current limiters, and superconducting magnetic energy storage [1,2,3,4,5,6,7]. At present, there are several main process methods to prepare REBCO films, such as pulsed laser deposition (PLD) [8,9], metal organic deposition (MOD) [10,11], sputtering [12], co-evaporation [13,14], and metal organic chemical vapor deposition (MOCVD) [15,16,17,18]. The PLD technology is a very mature method to prepare high-quality films [9,19]. However, its drawbacks include high equipment requirements; for example, the laser renders this technique very expensive to apply to industrial-scale productions. The MOD method has the advantages of low preparation cost and low equipment requirements. Yet, the quality of the film prepared by the MOD method is not high and its surface is relatively rough [10]. The sputtering method has the advantages of process stability, high film quality, and simple equipment requirements. Its disadvantage is that the deposition rate of the film is relatively slow [12]. The co-evaporation method can be also used to prepare REBCO films and its advantages include a high deposition rate and good process repeatability. Its disadvantage is high equipment requirements [13]. Because the MOCVD method has the advantages of low vacuum requirement, easy composition adjustment, and high production efficiency [18,20], a home-made MOCVD system was used to prepare GdYBCO HTS films by us. However, its disadvantage is that the necessary metal organic sources are too expensive. 

**Figure 1:**
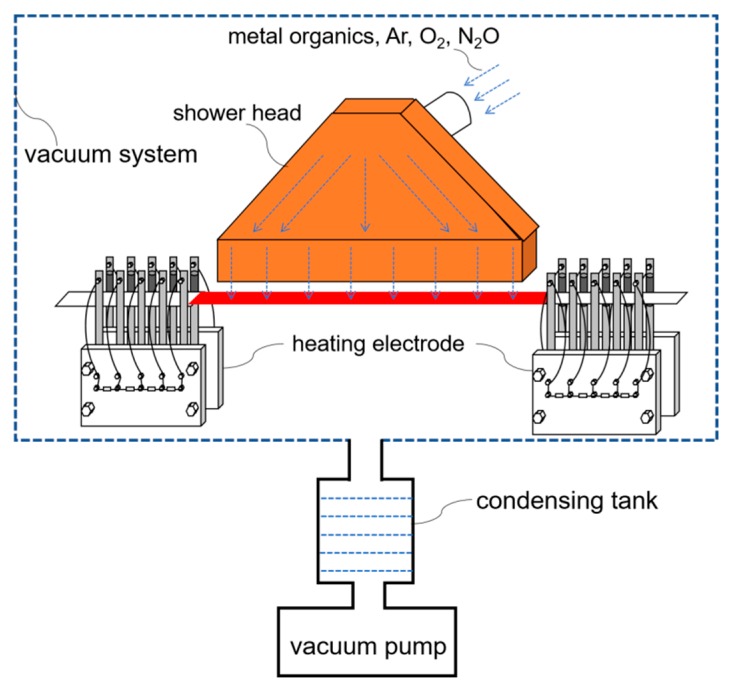
Schematic diagram of GdYBCO film preparation through using the self-heating technology.

In order to improve the utilization ratio of metal organic sources and reduce the cost, a novel self-heating method was adopted by us to heat the metal tapes in the MOCVD system [21], as is shown in Figure 1. The heating current was introduced into the metal tape by means of two home-designed electric brushes and the tape was heated by the Joule effect of self-resistance. Different from common radiation heating, the self-heating method does not cause excessive temperature increases of the shower head. Therefore, the shower can be very close to the surface of the heated metal tape. In this way, the growth rate of GdYBCO films and the utilization ratio of metal organic sources can be greatly improved. For the reported MOCVD system [15,16,17,18], in order to ensure the lateral uniformity of film preparation, the relatively large distance between the shower and the substrate needs to be maintained to provide enough time for the diffusion of metal organic sources. However, compared with other reported MOCVD systems, the shower in our system can be very close to the substrate, which can improve the deposition rate and the utilization ratio of metal organic sources. Therefore, when the shower head is very close to the tape surface, the shower head becomes the key component to uniformly deposit the GdYBCO superconducting films in the experimental chamber.

**Figure 2:**
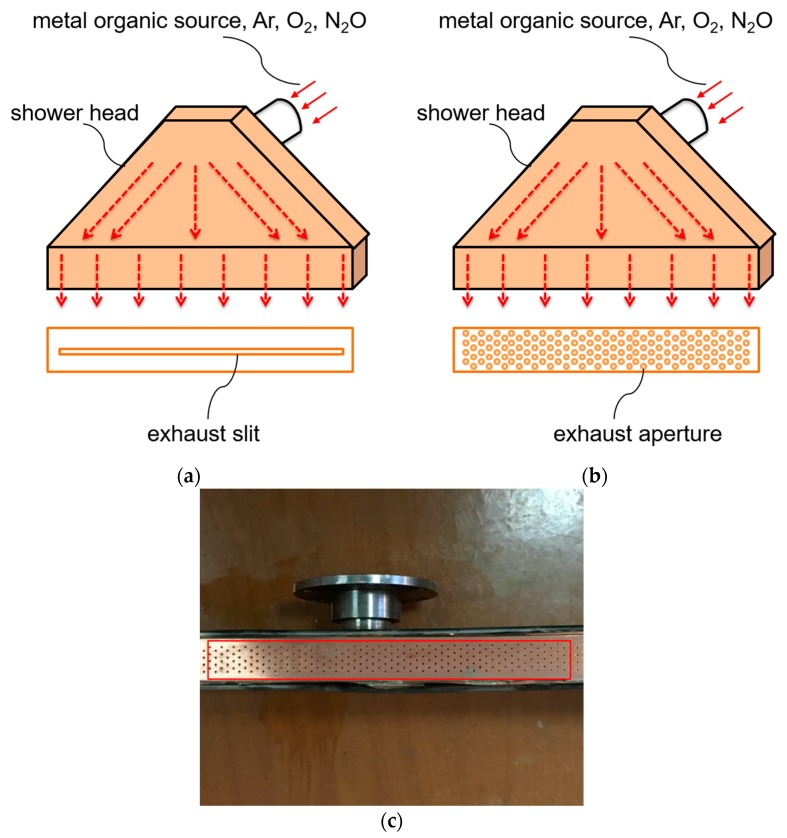
(**a**) The schematic diagram of the slit shower; (**b**) The schematic diagram of the multi-aperture shower; (**c**) A picture of the multi-aperture shower.

As is shown in Figure 2a,b, the 2-mm wide slit shower and the multi-aperture shower were used to prepare GdYBCO films based on the novel self-heating technology, respectively. The distance from the slit to the substrate surface is about 1 cm and the concentration of metal organic sources on the surface of substrate will be very high, which can greatly improve the deposition rate. However, because the distance from the nozzle to the tape is very small, the transverse uniformity of film based on the slit shower is poor. Therefore, through using the multi-aperture shower instead of the slit shower, the results of this work verify the feasibility of the multi-aperture shower application for the preparation of uniform GdYBCO films through using the self-heating technology. Thus, the uniform and rapid preparation of GdYBCO films can be achieved, which can improve the performance of production and reduce the cost.

## 2. Experiment

**Figure 3:**
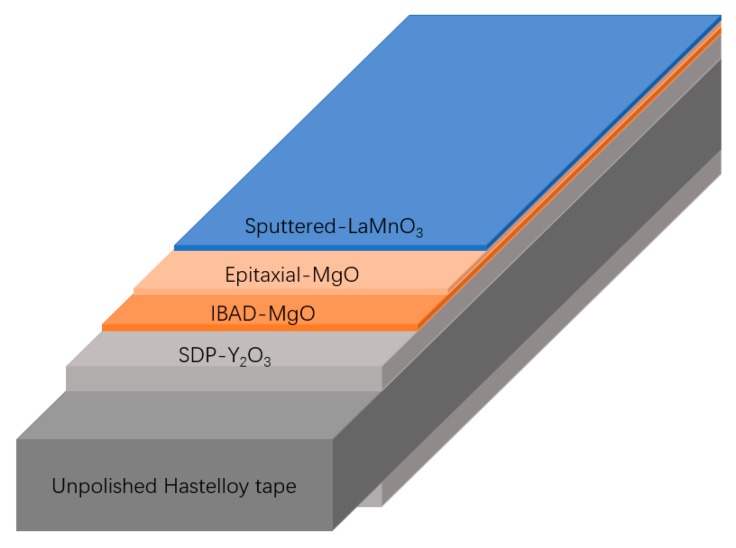
The schematic diagram of the buffer layers on the Hastelloy tape.

In our experiments, the GdYBCO films were prepared by the MOCVD system on the templates of sputtered-LaMnO_3_ (LMO)/homo-epitaxial MgO/IBAD (ion beam-assisted deposition)-MgO/SDP (solution deposition planarization)-Y_2_O_3_/Hastelloy tapes [22,23,24,25,26], as shown in Figure 3. The metal tapes were directly heated by self-resistance after applying a heating current. As shown in Figure 2, the 2-mm wide slit shower and the multi-aperture shower were used to deposit the GdYBCO films, respectively. The diameter of the apertures is 0.5 mm and the distance between the two adjoining apertures in the width direction is 2 mm, as shown in Figure 2b,c. The distance from the shower to the substrate surface is about 1 cm and the concentration of metal organic sources on the surface of substrate will be very high, which can greatly improve the deposition rate. Because the thickness of the metal tape is basically uniform, the resistance of the metal tape is certain. Thus, for a certain thickness of template tape, the surface temperature of the tape is decided by the heating current. The liquid precursor, which was prepared through dissolving the metal organic solids (MO sources, Samri Advanced Materials) of Zr(tmhd)_4_, Gd(tmhd)_3_, Y(tmhd)_3_, Ba(tmhd)_2_·(1,10-phenanthroline)_2_, and Cu(tmhd)_2_·(tmhd: 2,2,6,6-tetramethyl-3,5-heptanedionate) into tetrahydrofuran by the mole ratio of 0.06:0.6:0.6:2.0:2.2, was nebulized into an evaporator of 300–310 °C by the home-designed nozzle and evaporated quickly. Then, the evaporated vapor was mixed with the argon, oxygen, and nitrous oxide, of which the mass flow ratio was 2.44:1.06:1, and was reacted on the surface of the heated tapes to form GdYBCO films. Finally, the deposited GdYBCO films were annealed at 500 °C in an oxygen atmosphere.

**Figure 4:**
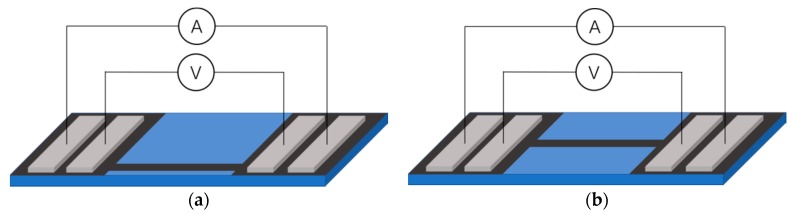
The schematic diagram of the four-probe method for the *I_c_* test of the GdYBCO micro-bridge: (**a**) the edge position; (**b**) the middle position.

The texture of the prepared GdYBCO films at the edge and middle positions was measured with *θ*–2*θ* scan, *ω*-scan, *φ*-scan, and *Chi*-scan through an X-ray diffraction system (XRD, Bede D1 system, BEDE, Durham, England). The surface morphology of the prepared GdYBCO films at the different positions was characterized by scanning electron microscopy (SEM, JEOL7500F, JEOL Ltd., Tokyo, Japan). Also, the composition of the thin films was characterized by energy dispersive spectrometry (EDS, Oxford INCA, Oxford Instruments, Oxford, England). The thickness was measured by a step profiler (Veeco Dektak 150, Veeco Instruments Inc., New York, NY, USA). Moreover, as is shown in Figure 4, the micro-bridge of the GdYBCO films of the middle and edge positions were prepared and tested, respectively. The critical current (*I_c_*) of GdYBCO micro-bridges at the edge and middle positions at 77 K and 0 T were obtained by the standard four-probe method using the criteria of 1 μV/cm, respectively.

## 3. Results and Discussion

### 3.1. The Preparation of GdYBCO Films Based on the Slit Shower

**Figure 5:**
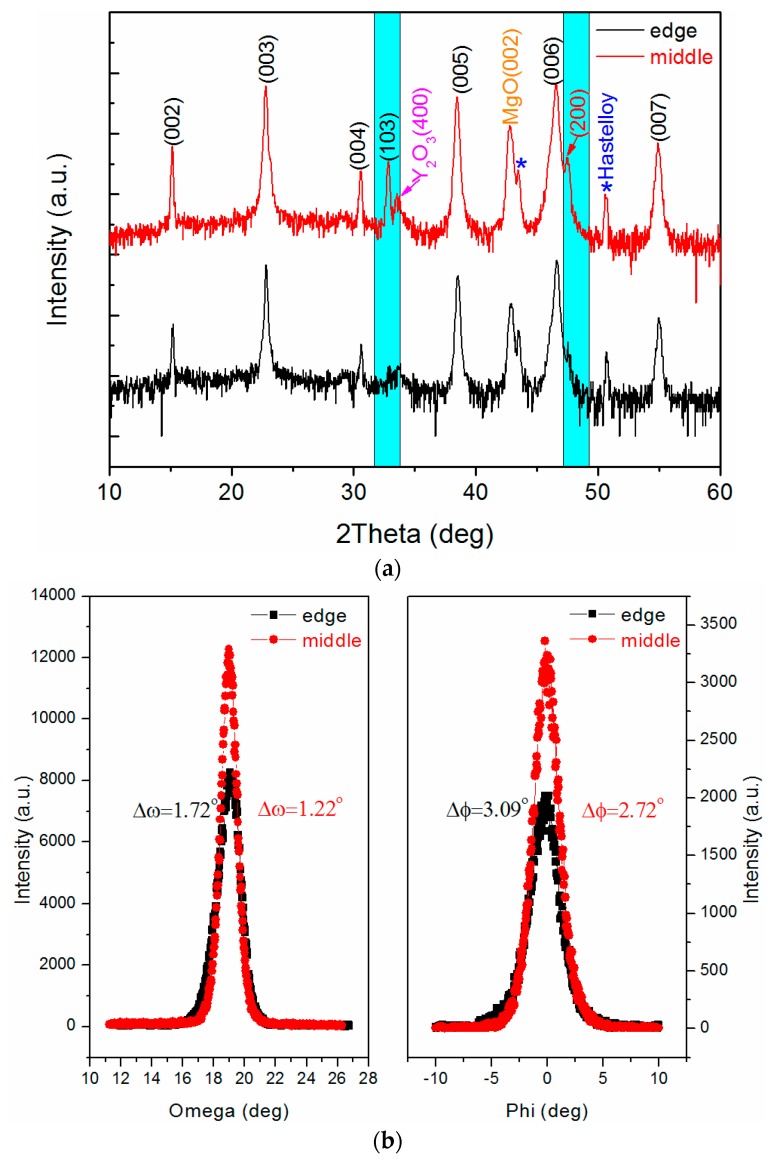
(**a**) The XRD *θ*–2*θ* scanning patterns of the GdYBCO samples prepared based on the slit shower; (**b**) The XRD *ω*-scan of GdYBCO (005) and the XRD *φ*-scan of GdYBCO (103).

Based on the slit shower, the edge and middle positions of the prepared GdYBCO samples were measured by the XRD *θ*–2*θ* scan, and the corresponding curves are shown in Figure 5a. Besides the diffraction peaks of the MgO layer and LaMnO_3_ layer, there are both the GdYBCO (00*l*) diffraction peaks and (*h*00) diffraction peaks in the XRD *θ*–2*θ* scanning curve of the middle position. This indicates that there are both the *c*-axis-oriented grains and *a*-axis-oriented grains at the middle position of the deposited GdYBCO films. However, compared with the (200) diffraction peak of *θ*–2*θ* scanning curve of the middle position, the (200) diffraction peak is obviously weakened in the XRD *θ*–2*θ* scanning curve of the edge position, which shows the inhomogeneity of the deposited GdYBCO films at the edge and middle positions. Meanwhile, the XRD *ω*-scan of GdYBCO (005) and *φ*-scan of GdYBCO (103) were carried out, and the measured results are shown in Figure 5b. As can be seen from the results, the full width at half maximum (FWHM) values of the *ω*-scan and *φ*-scan curves of the GdYBCO film at the middle position are 1.22° and 1.72°, respectively, and the FWHM values of the *ω*-scan and *φ*-scan curves of the GdYBCO film at the edge position are 1.72° and 3.09°, respectively. Similarly, it also clearly reflects that the texture of the prepared GdYBCO films in the transverse direction is not uniform based on the slit shower.

**Figure 6:**
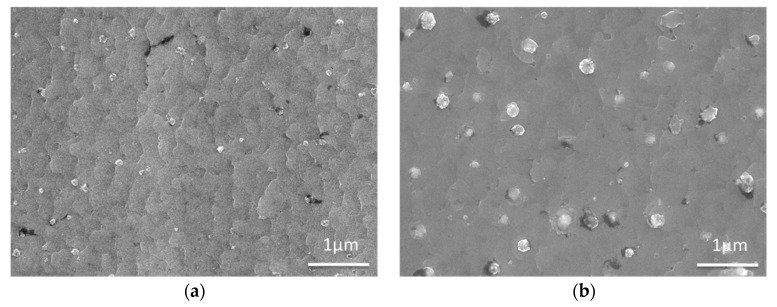
SEM images of the GdYBCO films prepared based on the slit shower: (**a**) SEM image of the edge position; (**b**) SEM image of the middle position.

The surface morphologies at the edge and middle positions are characterized by SEM, and the images are shown in Figure 6. As shown in Figure 6a, there is only a small amount of outgrowths on the surface of the film, and the size of impurities is relatively small. However, as shown in Figure 6b, there are some bigger outgrowths on the surface of the middle position of the GdYBCO film, and these outgrowths were revealed to be Ba-Cu-O phases by the EDS. Thus, it is shown that differences in the deposition condition in the transverse direction will lead to the inhomogeneity of the morphology of GdYBCO films.

**Figure 7:**
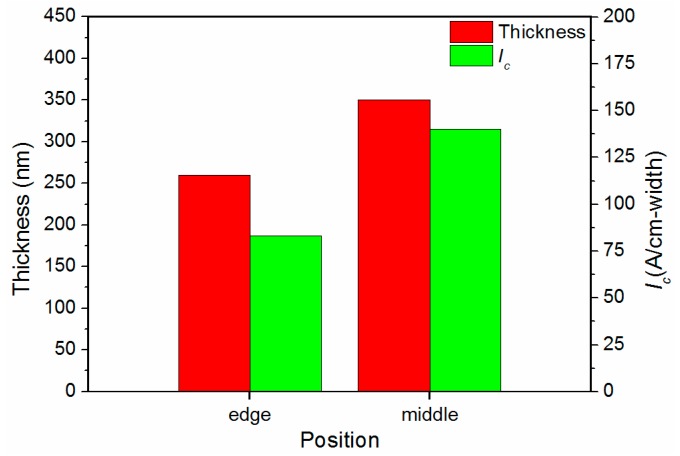
The thickness and *I_c_* histograms of the GdYBCO films prepared based on the slit shower.

The thickness of the deposited GdYBCO films at the edge and middle positions of the tape was measured by a step profiler, and the thickness values are shown in Figure 7. As can be seen from the histogram of thickness, the thickness of the middle position of the GdYBCO film is obviously thicker than that of the edge position, which indicates that the deposition rate of the GdYBCO superconducting film at the middle position is obviously higher than that of the edge position. Meanwhile, the *I_c_* of the middle position and edge position were measured by the four-probe method. As shown in Figure 7, the micro-bridge of the GdYBCO films of the edge and middle positions were tested, respectively. Figure 7 shows that the *I_c_* of the middle position is also obviously higher than that of the edge position. This indicates that the ability to carry current at the middle position is obviously stronger than that at the edge position in the transverse direction when the slit shower is used to prepare GdYBCO films.

### 3.2. The Preparation of GdYBCO Films Based on the Multi-Aperture Shower

**Figure 8:**
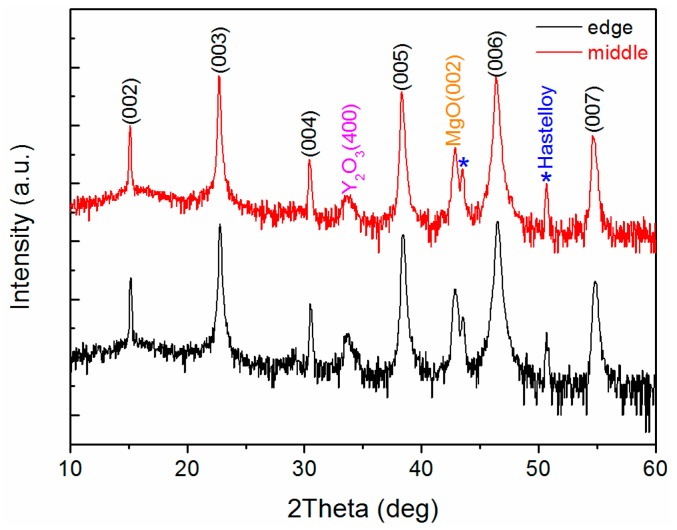
The XRD *θ*–2*θ* scanning patterns of the edge and middle positions of the GdYBCO samples prepared based on the multi-aperture shower.

Because the slit shower is too close to the surface of the buffered tapes, the vapor of metal organic sources cannot adequately spread in the transverse direction, which can lead to the different concentration distribution of metal organic sources in the transverse direction. Thus, the deposition rate of the GdYBCO films on the surface of the template will be not uniform when the slit shower is used in the MOCVD system. Therefore, the new home-made multi-aperture shower was used to prepare the GdYBCO films, which could complete an average allocation before the vapor of the metal organic sources flows out from the shower head. As shown in Figure 8, the edge and middle positions of the prepared GdYBCO samples were measured by the XRD *θ*–2*θ* scan and the corresponding curves are shown in Figure 8. Besides the diffraction peaks of the MgO layer and LaMnO_3_ layer, there are only the GdYBCO (00*l*) diffraction peaks, not (*h*00) diffraction peaks in the XRD *θ*–2*θ* scanning curves of the middle and the edge positions. This indicates that there are only the *c*-axis-oriented grains, not *a*-axis-oriented grains in the GdYBCO films. What is more, the intensity of the GdYBCO (00*l*) peaks of the middle and the edge positions is approximately the same, which indicates that the GdYBCO films at the middle and the edge positions exhibit little difference.

**Figure 9:**
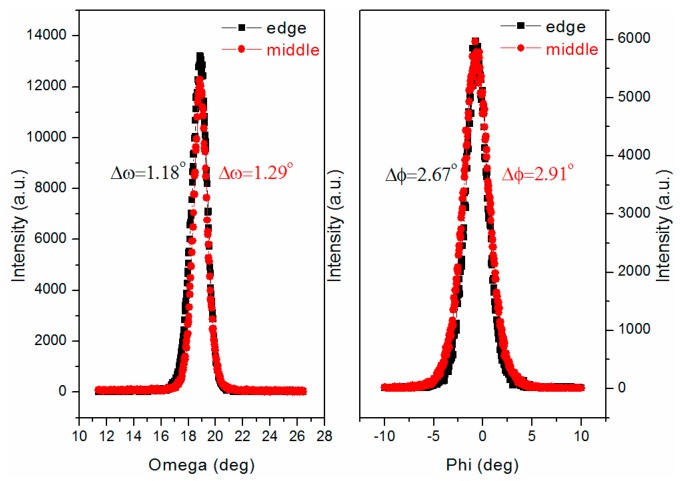
The XRD *ω*-scan of GdYBCO (005) and the XRD *φ*-scan of GdYBCO (103) based on the multi-aperture shower.

In order to characterize the biaxial texture of the edge and middle positions of GdYBCO films, the XRD *ω*-scan of GdYBCO (005) and *φ*-scan of GdYBCO (103) were carried out, and the measured results are shown in Figure 9. As can be seen from the results, the full width at half maximum (FWHM) values of the *ω*-scan and *φ*-scan curves at the middle position are 1.29° and 2.91°, respectively, and the FWHM values of the *ω*-scan and *φ*-scan curves at the edge position are 1.18° and 2.67°, respectively, which indicates that the textures of the GdYBCO superconducting films are very good at both the edge and middle positions.

**Figure 10:**
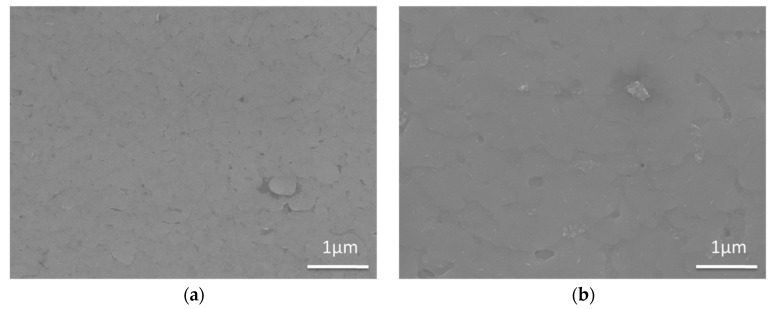
SEM images of GdYBCO films prepared based on the multi-aperture shower: (**a**) SEM image of the edge position; (**b**) SEM image of the middle position.

**Figure 11:**
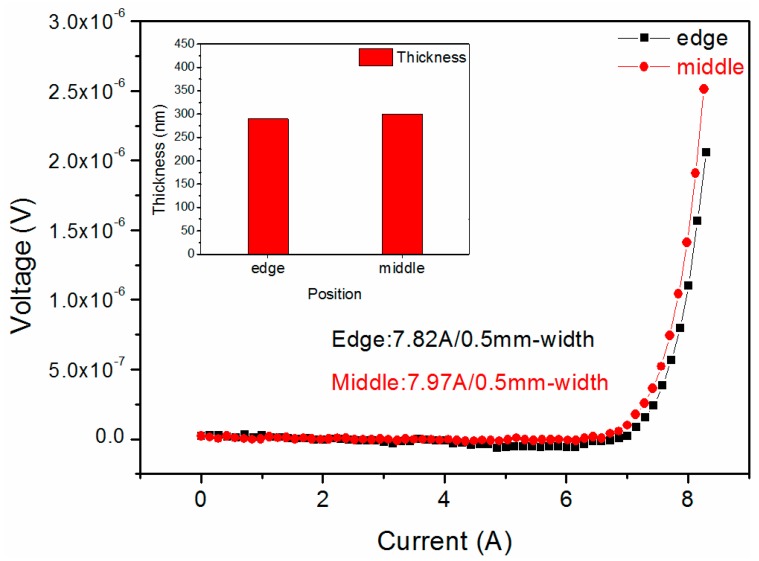
The thickness and *I_c_* histograms of GdYBCO films prepared based on the multi-aperture shower.

Moreover, the surface morphologies at the edge and middle positions were characterized by SEM, and the images are shown in Figure 10. Figure 10 shows that the surface of the middle and edge positions of GdYBCO films are dense and smooth, and there are no other outgrowths. Similarly, the thickness of the deposited GdYBCO films at different locations of the tapes was measured by a step profiler and the thickness values of the prepared GdYBCO film at the different positions are shown in Figure 11. Compared with the slit shower, the thickness uniformity of the GdYBCO films prepared by adopting the multi-aperture shower is greatly improved and the percentage of the maximum thickness difference is less than 5%. The *I_c_* of the middle and edge positions were measured by four-probe method, and the test curves are shown in Figure 11. Figure 11 shows that the transport critical current density (*J_c_*) of the middle and edge positions at 77 K and the self-field is over 5 MA/cm^2^ and exhibits little difference, which shows that the ability to carry current in the transverse direction is basically uniform, combining the thickness measurement results. Compared with the prepared GdYBCO films based on the slit shower, the performance nonuniformity of the deposited GdYBCO films based on the multi-aperture shower was greatly improved.

## 4. Conclusions

In this paper, the slit shower and the multi-aperture shower were used to prepare GdYBCO films on LaMnO_3_ templates based on the novel self-heating technology, respectively. The transverse uniformities of structure, morphology, and performance were characterized by X-ray diffraction system (XRD), scanning electron microscopy (SEM), and the standard four-probe method using the criteria of 1 μV/cm, respectively. The results show that the uniformity problem of GdYBCO film preparation can be well solved through the use of the new multi-aperture shower instead of the slit shower. Compared with the slit shower, the thickness uniformity of the GdYBCO films prepared by adopting the multi-aperture shower was greatly improved and the percentage of the maximum thickness difference was less than 5%. The transport critical current density (*J_c_*) of the middle and edge positions at 77 K and the self-field was over 5 MA/cm^2^ through adopting the multi-aperture shower. Therefore, based on the work, the effects of the diameter size of the single aperture and the apertures distribution on the structure, the residual stress [27], the performance, and the deposition rate can be studied in detail in future research.
